# 

**Published:** 1788

**Authors:** 


### THE LONDON MEDICAL JOURNAL,

FOR THE YEAR 1788,

PART THE SECOND.

### CATALOGUE OF BOOKS.

1 A DISSERTATION on the Properties of Pus; which gained the Prize Medal, given by the Lyceum Medicum Londinense, for the Year 1788, and was ordered to be printed for the Use of the Society. By *Everard Home*, F. R. S. and one of the Presidents of the Lyceum Medicum. 4to. *Richardson*, London, 1788.

2. Observations

2. Obſervations on the Diſeaſes of the Army in Jamaica; and on the beſt means of preſerving the Health of Europeans, in that Climate. By *John Hunter*, M. D. F. R. S. and Phyſician to the Army. 8vo. *Nicol*, London, 1788.

3. A Deſcription of all the Burſæ mucoſæ of the human Body; their Structure explained, and compared with that of the capſular Ligaments of the Joints, and of thoſe Sacs which line the Cavities of the Thorax and Abdomen; with Remarks on the Accidents and Diſeaſes which affect thoſe ſeveral Sacs, and on the Operations neceſſary for their Cure. Illuſtrated with Tables. By *Alexander Monro*, M. D. Profeſſor of Phyſic, Anatomy, and Surgery in the Univerſity of Edinburgh; Fellow of the Royal College of Phyſicians and of the Royal Society of Edinburgh; and Fellow of the Royal Academy of Surgery at Paris. Folio, *Elliot*, Edinburgh, 1788.

4. Experiments and Obſervations on animal Heat, and the Combuſtion of inflammable Bodies; being an attempt to reſolve theſe Phenomena into a general Law of Nature. By *A. Crawford*, M. D. F. R. S. L. and E. and Member of the Philoſophical Societies of Dublin and Philadelphia. The ſecond Edition with very very large Additions. 8vo. *Johnson*, London, 1788.

5. A Syftem of Surgery. By *Benjamin Bell*, Member of the Royal Colleges of Surgeons of Ireland and Edinburgh, one of the Surgeons of the Royal Infirmary, and Fellow of the Royal Society of Edinburgh. Illuftrated with copper-plates. 8vo. 6 vols. *Elliot*, Edinburgh, 1783-8.

6. The Generation of animal Heat inveftigated. With an Introduction, in which is an Attempt to point out, and afcertain, the elementary Principles and fundamental Laws of Nature, and apply them to the Explanation of fome of the moft interefting Operations and ftriking Appearances of Chemiftry. By *E. Peart*, M. D. 8vo. Gainfborough, 1788.

7. An Enquiry into the Nature, Caufes, and Cure of the Confumption of the Lungs: with fome Obfervations on a late Publication on the fame Subject. By *Michael Ryan*, M. D. and Member of the Royal Antiquarian Society of Edinburgh. 8vo. Dublin, 1787.

8. An Effay on the Treatment of Confumptions; in which the Caufes and Symptoms are confidered, and a new Mode of Treatment propofed. By *R. Charles*, Surgeon at Winchefter. 8vo. *Herdsfield*, London, 1787.

9. An

9. An Eſſay on the malignant, ulcerated ſore Throat; containing Reflections on its Cauſes and fatal Effects, in 1787. With a remarkable Caſe, accompanied with large Purple Spots all over the Body, a Mortification of the Leg, &c. &c. By *William Rowley*, M. D. Member of the Univerſity of Oxford, the Royal College of Phyſicians in London, &c. &c. To which are added, Animadverſions on the preſent Defects in treating the Diſorder, improved and ſucceſsful Methods of Cure, and an Account of a new Species of temporary Madneſs, &c. 8vo. *Nourſe*. London, 1788.

10. Elements of Medical Juriſprudence. 8vo. *Becket*, London, 1788.

11. Surgical Tracts: containing a Treatiſe upon Ulcers of the Legs; in which former Methods of Treatment are candidly examined, and compared with one more rational and ſafe, effected without Reſt and Confinement. Together with Hints on a ſucceſsful Method of treating ſome ſcrophulous tumours, and the mammary Abſceſs, and ſore Nipples of lying-in Women. The ſecond Edition reviſed, enlarged, and defended. To which are now added Obſervations on the more common Diſorders of the

Eye,

Eye, and on Gangrene. By *Michael Underwood*, M. D. 8vo. *Matthews*, London, 1788.

12. Cafes of the Hydrocele, with Obfervations on a peculiar Method of treating that Difeafe. To which is fubjoined a fingular Cafe of Hernia Veficæ urinariæ, complicated with Hydrocele; and two Cafes of Hernia incarcerata. By *T. Keate*, Surgeon Extraordinary to her Majefty, and Surgeon to their Royal Highneffes the Prince of Wales and Duke of York. 8vo. *Walter*, London, 1788.

13. An Effay on the Bite of a mad Dog: with Obfervations on John Hunter's Treatment of the Cafe of Mr. R. and alfo a Recital of the fuccefsful Treatment of two Cafes. By *Jeffe Foot*, Surgeon. 8vo. *Becket*, London, 1788.

14. A few Remarks upon the Treatment and Cure of venereal and fcorbutic Diforders; fubmitting a new Medicine to the Confideration and the Experience of the Public: to which are added, feveral felect Cafes to prove the efficacy of the Remedy here recommended. By *J. Donovan*, Surgeon. 8vo. *Egerton*, London, 1788.

15. Curfory Remarks on the new Pharmacopœia. By Liquor volatilis Cornu Cervi. 8vo. *Stalker*, London, 1788.

16. An

16. An Eſſay on Crookedneſs, or Diſtortions of the Spine in Children; ſhewing the inſufficiency of a Variety of Modes made Uſe of for Relief in theſe Caſes: and propoſing Methods, eaſy, ſafe, and more effectual for the Completion of their Cures; with ſome Hints for the Prevention of theſe Affections, and their diſagreeable, painful, and dangerous Conſequences. Illuſtrated with ſeveral Copper-plates, taken from diſtorted ſubjects. By *Philip Jones*. 8vo. *Cadell*, London, 1788.

17. A Treatiſe on Medical and Pharmaceutical Chymiſtry; and the Materia Medica. To which is added, an Engliſh Tranſlation of the new Edition of the Pharmacopœia of the Royal College of Phyſicians of London, of 1788. By *Donald Monro*, M. D. Phyſician to the Army, and formerly to St. George's Hoſpital, Hyde Park Corner; Fellow of the Royal College of Phyſicians, and of the Royal Societies of London and Edinburgh. 8vo. 3 volumes. *Cadell*, London, 1781.

18. Diſſertatio Medica Inauguralis de Hepatitide Indiæ Orientalis. Auctore *Thoma Girdleſtone*, Anglo. 4to. Lugd. Batav. 1787.

19. Index Plantarum quas in agro Erfurtenſi ſponte provenientes olim *J. Phil. Nonne*, deinde deinde *J. J. Planer*, collegerunt. 8vo. Gothæ, 1788.

20. Synopſis Syſtematica Scriptorum, quibus inde ab Inauguratione Academiæ Georgiæ Auguſtæ d. 17 Sept. 1737, uſque ad ſollemnia iſtius Inaugurationis ſemiſæcularia 1787, Diſciplinam ſuam augere & ornare ſtuduerunt Profeſſores Medici Gœttingenſes. Digeſſit & edidit *Jo. Fr. Blumenbach*. 4to. Gœttingæ, 1788.

21. De Probabilitate Vitæ ejuſque Uſu forenſi, Commentatio prior, qua maxime Theoriam Expectationis Vitæ Antiquitati vindicat, *Frid. Aug. Schmelzer*. 8vo. Gœttingæ, 1788.

22. *Trnka de Krzowitz*, Hiſtoria Rachitidis, omnis ævi obſervata medica continens. 8vo. Vindobonæ, 1787.

23. *Trnka de Krzowitz*, Hiſtoria Timpanitidis, omnis ævi obſervata medica continens. 8vo. Vindobonæ, 1787.

24. Obſervationes Botanicæ circa Syſtema Vegetabilium Divi *a Linné*, Gœttingæ 1784 editum; quibus accedit juſtæ in manes Linneanos pietatis ſpecimen. Auctore *Andrea Dahl*, Weſtgothia-Sveco. 8vo. Havniæ, 1787.

25. Diſſertatio Epiſtolaris circa Inventionem Pulſus Antiſdicroti tamquam veri ſigni futuram Diarrhœam ventoſam demonſtrantis in proſecutionem inventi pulsifici Solaniani ad Regiam Academiam Medicam Londinensem a D. *Francisco Xaverio Cid*, Regiæ Societatis Cantabricæ Amicorum Patriæ Socio, Academiæ Medicæ Matritensis Academico, & Illustrissimi Decani & Capituli Sanctæ Toletanæ Ecclesiæ Hispaniarum Primatis, Excellentissimique & Illustrissimi D. D. Francisci Antonii Lorenzana, Archiepiscopi Toletani Medico. 8vo. Toleti, 1787.

26. Constitutionis ævi nostri febrilis quædam momenta. Consentiente illustri Medicorum Ordine summos in Medicina & Chirurgia honores ambiturus conscripsit *Albertus Rengger*, Helvetus. 8vo Gœttingæ, 1788.

27. Specimen Bibliothecæ criticæ Magnetismi sic dicti Animalis ; Consensu illustris Medicorum Ordinis pro obtinendis summis in Medicina & Chirurgia honoribus scripsit *Paulus Usteri*, Tigurino-Helvetus. 8vo. Gœttingæ, 1788.

28. *Thomæ Lauth*, M. D. Anat. & Chir. P. P. O. Nosologia Chirurgica. Accedit notitia Auctorum recentiorum Platnero. In Usum Prælectionum Academicarum. 8vo. Argentorati, 1788.

29. Oratio Inauguralis habita in Gymnasio Patavino 3 Id. Octobr. An. 1786 a *Stephano Gallino* cum primùm ad Theoricam Medicinam Ordinariam publice profitendam accederat. 4to. Venetiis, 1787.

30. Analyse des Eaux Minerales de Charbonnière dites de Laval; par M. *de Marsonnat*, Curé de la Paroisse de Tassin & Charbonnière, en Lyonnois. 8vo. Lyon, 1787.

31. Précis du Succès des Eaux Minerales de Charbonnière, dites de Laval; par M. *de Marsonnat*. 8vo. Lyon, 1787.

32. Description des Bains de Geismar en Hesse; par un Ami de l'Humanité. 8vo. Berlin, 1787.

33. Avis au Peuple François sur sa Santé, ou Précis de Médecine pratique, propre aux différens Lieux, Temps, Circonstances, & au Temperament de la Nation; par M. *de Lavaud*, ancien Chirurgien-Major dans les Armées navales, 8vo. Paris, 1788.

34. Traité de l'Insertion de la petite Verole, ou de l'Inoculation, reduite, d'après un grand nombre d'Observations, à l'Etat de Simplicité qu'elle exige pour être infailliblement salutaire; par M. *Tudesq* fils, Docteur en Médecine de l'Université

l'Univerſité de Montpellier, Médecin en Chef de l'Hôpital Militaire de la Ville de Cette, &c. 8vo. Montpellier, 1787.

35. Extrait des Regiſtres de l'Academie Royale des Sciences, du 12 Mars 1788. Troiſième Rapport des Commiſſaires chargés, par l'Academie, des Projets relatifs à l'Etabliſſement des quatre Hôpitaux. Imprimé par Ordre du Roi. 4to. Paris, 1788.

This Report contains the Obſervations made by two of the Commiſſioners (Meſſieurs Tenon and Coulomb) on the Engliſh Hoſpitals; and likewiſe a Plan and Deſcription of the four Hoſpitals intended to be erected at Paris.

36. Verſuch über den Nutzen der Nervenknoten, *i. e.* An Eſſay on the Uſes of the Ganglions of the Nerves. By *James Johnſtone*, M.D. Tranſlated from the Engliſh. 8vo. Stettin, 1787.

37. Ein Paar Worte über die Pocken und über die Inoculation derſelben. *i. e.* A Word or two on the ſmall Pox and on Inoculation. By *C. F. Elſner*, M. D. Profeſſor of Phyſic at Koningsberg. 8vo. Koningsberg, 1787.

38. Neue Bemerkungen und Erfahrungen zur Bereicherung der Wundarzneykunſt und

Arzneygelahrheit.

Arzneygelahrheit. *i. e.* New Obſervations and Experiments for the Improvement of Surgery and Phyſic. By *O. J. Evers.* 8vo. Gœttingen, 1787.

39. Kleine Phyſikaliſch-chemiſche Abhandlungen. *i. e.* Phyſico-chemical Eſſays. By *John Fred. Weſtrumb.* vol 2d. 8vo. Leipſic, 1787.

40. Verſuch einer vollſtændigen Abhandlung über die ſo genannte Engliſche Krankheit. *i. e.* An Eſſay towards a compleat Treatiſe on the ſo called Engliſh Diſeaſe. By *J. F. L. Cappel,* M. D. Aſſeſſor of the Imperial College of Phyſicians at Peterſburgh. Part. I. 8vo. Berlin, 1787.

41. Von Thieriſchen Magnetiſmus. *i. e.* On Animal Magnetiſm. By *E. Gmelin.* 8vo. Tubingen, 1787.

42. Chemiſche Abhandlung vom Schwefel. *i. e.* A Chemical Treatiſe on Sulphur. By *F. Aug. von Waſſerberg.* 8vo. Vienna, 1787.

43. Memoria Iſtorica della Febbre Epidemica, che ebbe corſo nella Terra di S. Stephano Ducato di Milano dal Principio di Ottobre dell' Anno 1783 fino al compierſi di Giugno del 1784 dell Dott. *Franceſco Beretta*

Medico

Medico nel Borgo di Magenta, e Socio delle Academie di Botanica, e de Georgofili di Firenze. 8vo. Milano, 1787.

44. Malattia verminosa della vescica, descritta dal Sig. Dott. *Jacopo Panzani.* 8vo. Venizia, 1787.

This is the case of a man, fifty years old, who after having for some time laboured under a painful complaint, which was thought to be occasioned by a stone in the Bladder, was relieved by voiding two small round worms (*lumbrici*) with his urine.

45. Saggio Medico sui vasi linfatici, &c. Coi mezzi di prevenire gli effetti delle sostanze velenose, come sarebbe la saliva del cane arrabbiato, il veleno della vipera, il veleno venereo, &c. Del Sig. *Assalini* il figlio. 8vo. Torino, 1787.

46. Memoria per servire all' intiera perfetta estinzione in tutte le Nazioni Europee del vajuolo, e di tutti i morbi contagiosi si acuti che cronici eccettuatane la lue venerea, &c. Del Sacerdote D. *Francesco Maria Scuderi*, di Via grande in Sicilia, Dottore di Medicina, tradotta dal latino dallo stesso Autore. 8vo. Napoli, 1787.

47. Recueil

47. Recueil d'Obſervations, ou Mémoire ſur l'Epidémie qui a regné en 1784 & 1785 dans la Subdélégation de la Châtaigneraye, en bas Poitou; ſuivi d'un Supplément ſur les Maladies regnantes pendant l'année 1786; accompagné de Notices ſur les mêmes Maladies dans les différens Départemens de la Généralité de Poitiers; extraites de la Correſpondance de M. *Pallu*, Conſeiller du Roi, Doyen, Docteur-Régent de la Faculté de Médecine en l'Univerſité de Poitiers; Médecin en chef des Epidemies du Poitou, Correſpondant de la Société Royale de Médecine de Paris: Ouvrage qui a remporté un des premiers prix de la Société Royale, le 29 Août 1785, publié par Ordre du Gouvernement & aux frais du Roi; par M. *J. G. Gallot*, Docteur en Médecine de l'Univerſité de Montpellier, Médecin de S. A. S. Monſeigneur le Duc d'Orléans, Conſeiller-Médecin-ordinaire du Roi, &c. 4to. Poitiers, 1787.

48. Eſſai ſur l'Hiſtoire Naturelle de la Groſſeſſe & de l'Accouchement. Par M. *Alphonſe le Roy*, Docteur-Régent, Profeſſeur de Médecine, des Accouchemens, & ancien Profeſſeur de Chirurgie des Ecoles de la Faculté de Médecine de Paris. 8vo. Paris, 1787.

